# Changes in S-100 protein serum levels in survivors of out-of-hospital cardiac arrest treated with mild therapeutic hypothermia: a prospective, observational study

**DOI:** 10.1186/cc7785

**Published:** 2009-04-16

**Authors:** Matthias Derwall, Christian Stoppe, David Brücken, Rolf Rossaint, Michael Fries

**Affiliations:** 1 Department of Anaesthesiology, University Hospital RWTH Aachen, Pauwelsstrasse 30, 52074 Aachen, Germany; 2 Institute of Neuropathology, University Hospital RWTH Aachen, Pauwelsstrasse 30, 52074 Aachen, Germany

## Abstract

**Introduction:**

Knowledge about the influence of current neuroprotective interventions on prognostic markers after survival from cardiac arrest is lacking. This study aimed to investigate the effects of mild therapeutic hypothermia on the release of the astroglial protein S-100 after cardiopulmonary resuscitation (CPR) in survivors of out-of-hospital cardiac arrest.

**Methods:**

This was a prospective, observational study performed during a two-year period, involving medical emergency services and five collaborating hospitals at the city of Aachen, Germany. Sixty-eight subjects were enrolled by the emergency physician on duty by taking blood samples after successful attempts at resuscitation with return of spontaneous circulation (ROSC), followed by samples at 6, 12, 24, 72 and 120 hours post ROSC by the appropriate intensive care unit staff. Depending on the decision of the attending physician, subjects were cooled down to 33°C (n = 37) for 24 hours or were held at 37°C (n = 31). Patients were tracked for estimating mortality and gross neurological outcome for 14 days.

**Results:**

S-100 levels in patients not receiving mild therapeutic hypothermia (normothermia (NT)) showed equivalent numbers as compared with cooled patients (mild therapeutic hypothermia (MTH)) on baseline (NT = 1.38 μg/l versus MTH = 1.30 μg/l; *P *= 0.886). S-100 levels on baseline were significantly lower in patients with a good neurological outcome at 14 days after the event in comparison to their peers with adverse outcome (*P *= 0.014). Although the difference in S-100 levels of MTH patients with adverse or favourable neurological outcome reached statistical significance, it did not in NT patients.

**Conclusions:**

Although the predictive power of S-100 levels were best on admission but not at later time points, MTH had no influence on S-100 serum levels in survivors of non-traumatic out-of-hospital cardiac arrest in the particular setting of this investigation.

## Introduction

Sudden cardiac arrest (SCA) is the leading cause of death in the USA and Europe affecting about 750,000 people annually [1,2]. Because of improved public training of cardiopulmonary resuscitation (CPR) and advances in professional emergency medical response [3], the rate of return of spontaneous circulation (ROSC) has risen in the past decades. However, depending on the duration of the arrest, neurological survival is still a major concern [4]. The application of mild therapeutic hypothermia (MTH) has been demonstrated to significantly reduce neurological damage in survivors of SCA in two randomised controlled trials [5,6].

Clinically, it is desirable to rely on an early and specific marker for final neurological outcome. Protein S-100B is a potential candidate for estimating post hypoxic neuronal damage due to its neuronal specificity and characteristic behaviour in serum depending on the degree of damage to the central nervous system (CNS) [7-16]. Increased serum levels of S-100 also have prognostic value for unfavourable neurological outcomes in patients with traumatic brain injury, stroke and cardiac surgery [17-20]. Several studies have investigated its potential role as a prognostic marker in survivors of SCA and found it to be a reliable marker for hypoxic/ischaemic CNS damage [8,11,13,14,21,22]. The progressive implementation of MTH into clinical practice and its proven impact on neurological outcome has raised the question about its influence on serum levels of S-100.

The present study was therefore conducted to elucidate the influence of MTH on S-100 serum levels in survivors of non-traumatic out-of-hospital cardiac arrest.

## Materials and methods

During a two-year period from 2005 to 2007, 68 patients (aged over 18 years) suffering from non-traumatic out-of-hospital cardiac arrest were included in this prospective study. Severe pre-existing conditions diagnosed in the past six months including sepsis, stroke, previous CPR and cancer were regarded as exclusion criteria.

CPR was performed in accordance to European Resuscitation Council's guidelines for advanced life support 2000 [23], which were gradually replaced by the 2005 edition [24] during the investigation period. In general, professional emergency medical technicians were supervised by an emergency physician on scene.

Demographic and CPR-related data were collected at the emergency department immediately after hospital admission and after 6, 12, 24, 72 and 120 hours at the intensive care unit via a web-based data entry system using an Utstein-Style like template, introduced by the German Society of Anaesthesiology and Intensive Care Medicine [25]. Hospital admission and first withdrawal of blood was defined as baseline.

At corresponding time points, information about haemodynamic and metabolic parameters, such as heart rate (HR), mean arterial pressure (MAP) and lactate and glucose levels, as well as the proof of microbiological pathogens and whether catecholamines were used or not, were documented. Data about the time when MTH was started and how long MTH was maintained were also collected. The decision to initiate MTH was solely at the discretion of the attending physician.

At day 14, neurological outcome was assessed using the cerebral performance categories (CPC) by a physician unaware of the study. CPC 1 and 2 were regarded as favourable neurological outcome, whereas CPC 3 to 5 signified adverse outcome [26].

Because all personal data were kept anonymous and no additional blood samples were taken, the local ethics committee approved the study without the requirement to obtain informed consent from each patient.

Patients received standardised intensive care treatment including mechanical ventilation, tight glucose control, infection control and vasopressor treatment to maintain MAP above 65 mmHg. Additional interventions, such as heart catheterisation, were performed if necessary.

If it was decided to cool the patient, no active warming was applied before induction of MTH. Hypothermia was induced via infusion of one to two litres of cold (about 6°C) saline in combination with body surface cooling using bags filled with ice water. To avoid shivering, patients received a continuous intravenous infusion of non-depolarising neuromuscular-blocking drugs such as rocuronium or pancuronium. Although no specific instructions were supplied by the study protocol, the vast majority of patients nevertheless received a combined continuous infusion of either midazolam or propofol and an opioid. Caregivers were advised to cool down patients as fast as possible in the induction period and to aim to achieve a core temperature of 33°C for 24 hours and to rewarm the patient carefully, not exceeding 1°C per hour. Core temperature was measured with an oesophageal temperature probe, and rewarming was usually performed with a convecting heating blanket.

Serum samples for the determination of S-100 protein were taken from the supernatant of blood collected for routine laboratory analyses and stored at -80°C for later analysis. Serum levels were quantified using a commercially available automated system (LIAison, DiaSorin, Dietzenbach, Germany).

To detect influences of MTH on S-100 protein levels at the given time points, patients were grouped into those receiving MTH or normothermia (NT). In a second analysis, this data were evaluated regarding differences in the final CPC scores, that is, favourable vs. adverse neurological outcome, as defined above. Data were analysed using statistical software SPSS 14.0 (SPSS Inc., Chicago, IL, USA). All results are expressed as mean ± standard deviation. To establish differences between groups, analysis of variance was performed and corrected for multiple comparisons (Bonferroni) in the case of continuous variables. To detect changes over time, repeated measures analysis of variance was employed and followed by pairwise *t*-tests. Categorical data were analysed using chi-squared test. *P *< 0.05 was considered to indicate statistical significance.

## Results

No differences between patients treated with or without MTH were found with regard to most of the demographic and arrest-related data (Table 1). Patients treated with MTH were significantly more prone to bacterial infection and more often required catecholamines. Nevertheless, these patients also tended to have a higher in-hospital survival rate (MTH = 78.4% vs. NT = 54.8%; *P *= 0.067) accompanied by a slightly more favourable neurological outcome in comparison with the NT group (CPC ≤ 2: MTH = 56.8% vs. NT = 45.2%; *P *= 0.341). Grossly, haemodynamic and metabolic changes were comparable between groups (Data not shown).

**Table 1:** Demographical and clinical variables of patients treated with mild therapeutic hypothermia (MTH) or normothermia (NT)

	MTH (n = 37)	NT (n = 31)	*P*
Age, years	62.6 ± 15.7	67.1 ± 14.0	0.229
Sex, % male	64.7	74.2	0.442
Survival time, hours	120 ± 42	115 ± 24	0.914
Survivors, %	78.4	54.8	0.067
Good neurological outcome, %	56.8	45.2	0.341
Call-response-interval, min:sec	03:19 ± 02:26	03:05 ± 01:57	0.660
Total adrenaline, mg	4.3 ± 3.8	3.2 ± 3.8	0.286
Total shocks, n	3.5 ± 2.6	3.8 ± 5.7	0.760
Arrest witnessed, %	40.5	51.6	0.411
Initial rhythm VF, %	66.7	51.6	0.220
Cardiac origin of arrest, %	91.9	64.5	0.052
Proof of any pathogen, %*	67.6	12.9	0.001
Catecholamines at any time, %*	97.3	71.0	0.002
Core temperature at BL, °C*	35.5	36.4	0.011
Glasgow coma scale at BL	3 ± 2	4 ± 3	0.339

Results presented as mean ± standard deviation. * *P *< 0.05 MTH vs. NT. BL = baseline; VF = ventricular fibrillation.

**Figure 1:**
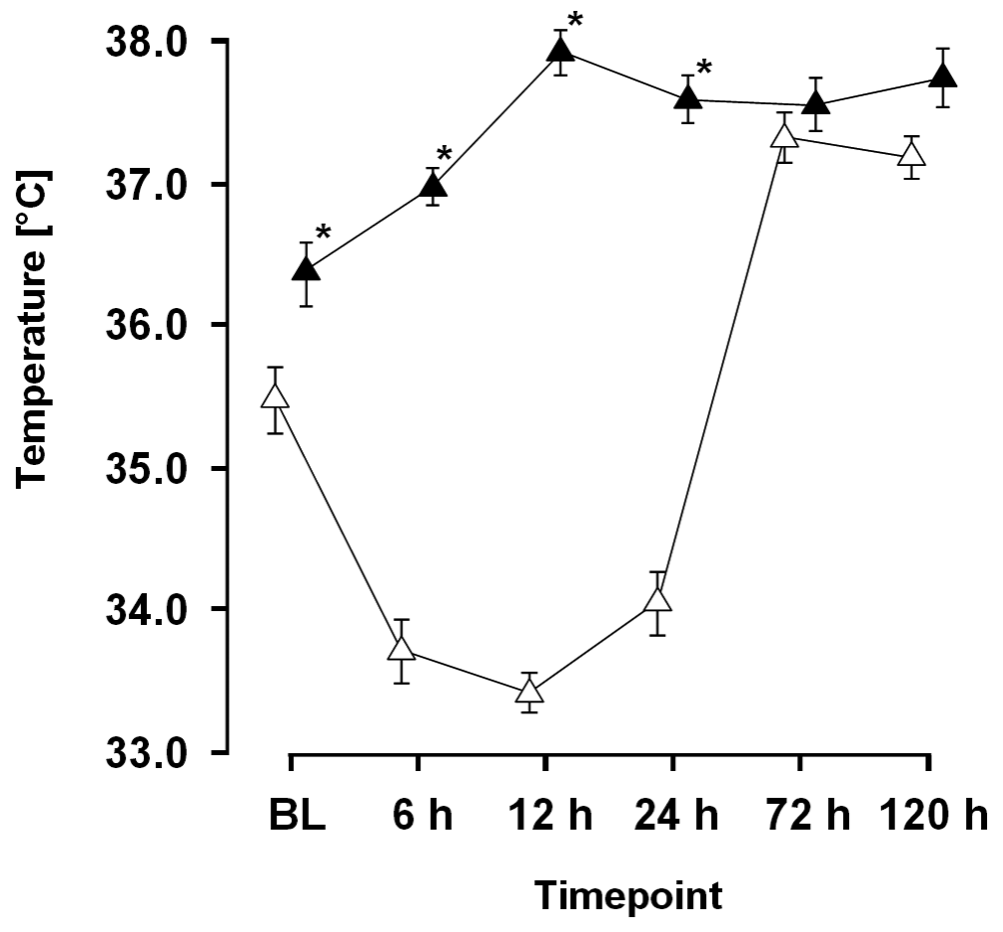
Time course of patient's oesophageal temperature. * *P *< 0.05 for mild therapeutic hypothermia vs. normothermia. BL = baseline.

Patients treated with MTH had significantly lower oesophageal temperatures already at baseline when compared with NT patients (35.5°C vs. 36.4°C; *P *= 0.011). The target temperature of 34°C was reached within 3.0 ± 2.2 hours after hospital admission and was maintained for 24.8 ± 4.9 hours. Lowest values were recorded 12 hours after baseline, with mean values of 33.4 ± 0.8°C. NT patients developed sub-febrile temperatures with a peak of 37.9°C at 12 hours post resuscitation (Figure 1).

S-100 levels at baseline were significantly elevated in patients with adverse neurological outcome (*P *= 0.014). This was also true after 24 and 72 hours (Table 2).

**Table 2:** S-100 Serum levels on each timepoint

Timepoint	Neurological outcome		*P *value
		
	Good (CPC 1 to 2)	Bad (CPC 3 to 5)	
BL*	0.81 ± 1.09	1.93 ± 1.78	0.028
6 hours	0.47 ± 0.45	0.71 ± 0.67	0.192
12 hours	0.37 ± 0.39	0.57 ± 0.53	0.166
24 hours*	0.27 ± 0.22	0.51 ± 0.31	0.002
72 hours*	0.21 ± 0.15	0.41 ± 0.40	0.030
120 hours	0.17 ± 0.09	0.19 ± 0.10	0.669

Results presented as mean ± standard deviation. * *P *< 0.05 cerebral performance categories (CPC) 1 to 2 vs. CPC 3 to 5. BL = baseline.

**Figure 2:**
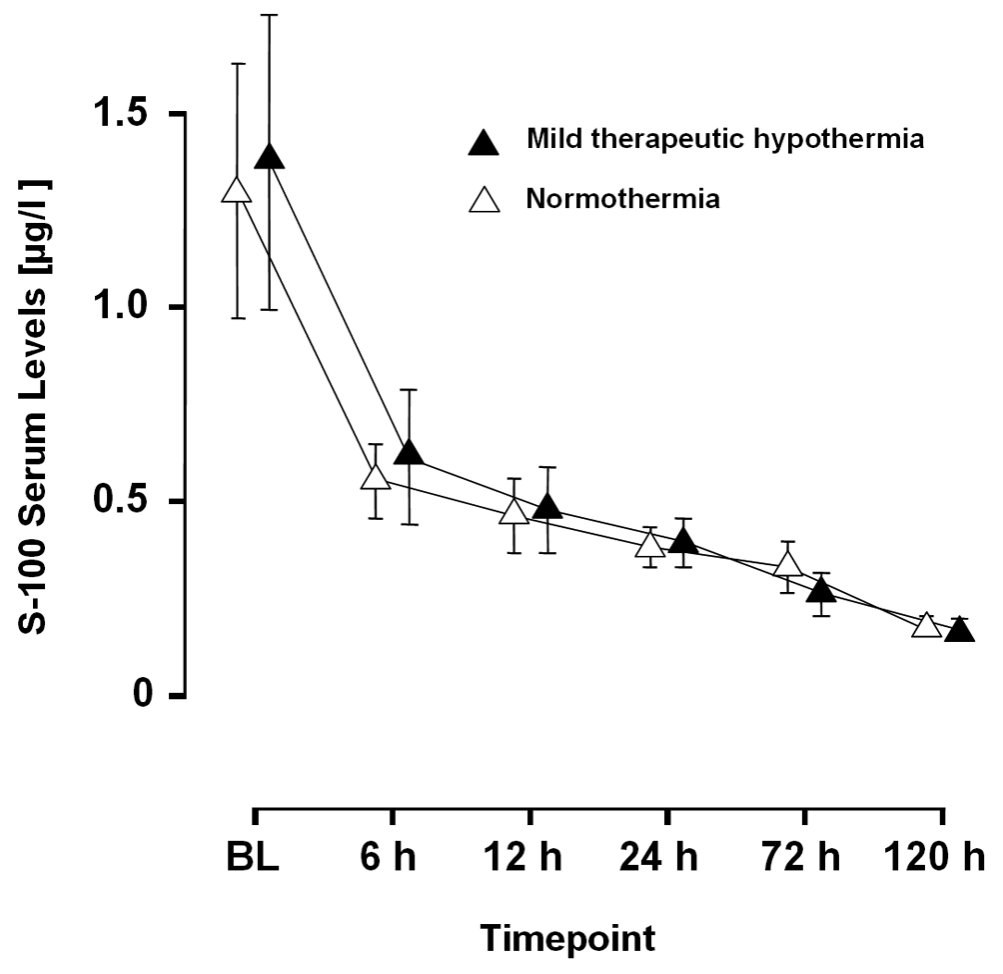
Time course of S-100 protein. S-100 protein serum levels in patients receiving mild therapeutic hypothermia (MTH) vs. normothermia (NT). BL = baseline.

There were no significant differences in S-100 serum levels between NT and MTH patients at any time point (Figure 2).

**Figure 3:**
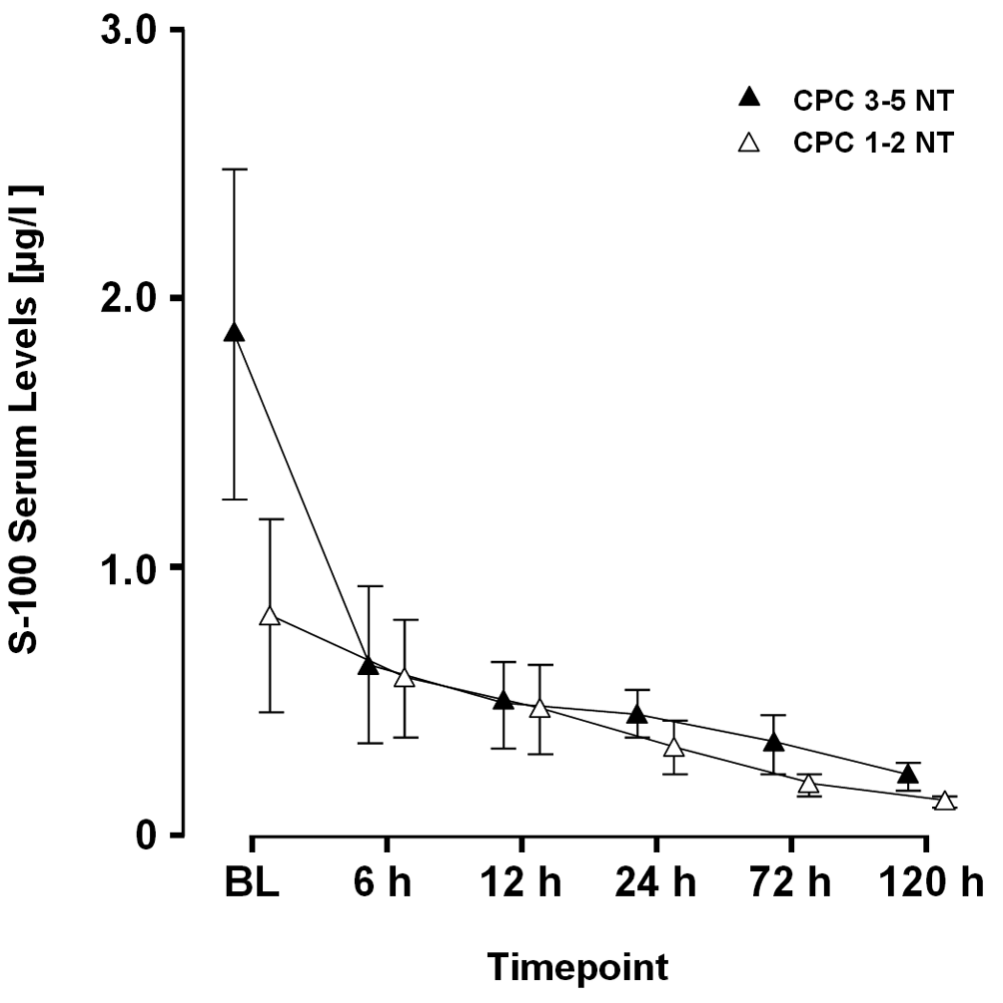
S-100 time course – normothermia. S-100 serum levels in patients (n = 31) receiving normothermia (NT) for cerebral performance categories (CPC) 1 to 2 vs. 3 to 5. BL = baseline.

**Figure 4:**
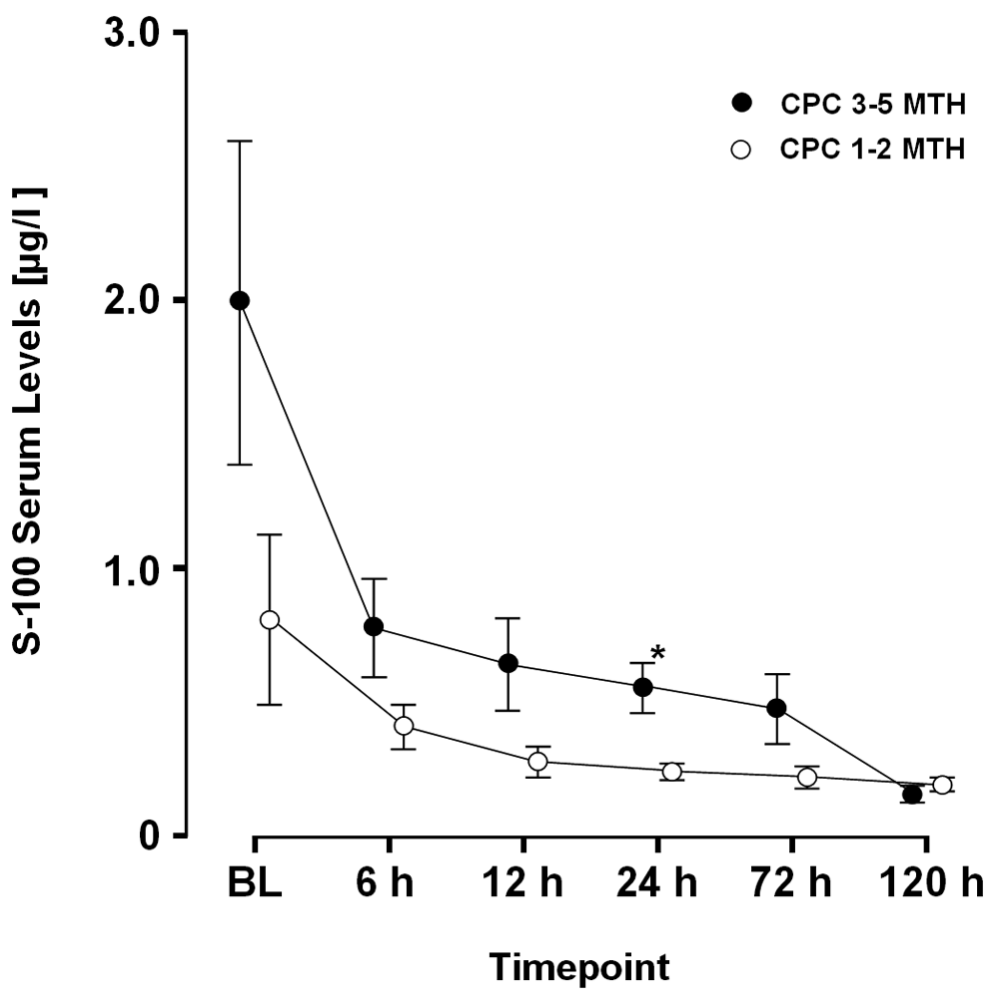
S-100 time course – mild therapeutic hypothermia. S-100 serum levels in patients (n = 37) receiving mild therapeutic hypothermia (MTH). * *P *< 0.05 for cerebral performance categories (CPC) 1 to 2 vs. 3 to 5. BL = baseline.

Regardless of neurological outcome, S-100 serum levels were almost congruent from six hours after ROSC in NT patients as depicted in Figure 3. In contrast, patients treated with MTH and a favourable neurological outcome showed a strong trend to lower S-100 serum levels being significant after 24 hours (CPC 1 to 2 = 0.56 vs. CPC 3 to 5 = 0.24; *P *= 0.001; Figure 4).

## Discussion

In the present study the administration of MTH did not significantly influence serum levels of S-100 protein in patients surviving non-traumatic out-of-hospital cardiac arrest. Both patients treated with or without MTH showed comparable decreases in S-100 serum levels over time (Figure 2). These findings were only marginally different when patients were stratified according to the final neurological outcome.

S-100 protein is an astrocyte-derived neurotrophic protein which is strongly associated with the promotion of neuronal growth and survival [27]. It is predominately found in astrocytes and Schwann cells [28] and may play a crucial role in the process of learning and memory [29]. Due to its molecular weight of 21,000 Dalton, S-100B may only be detected in peripheral blood if the integrity of the blood-brain barrier (BBB) is disrupted. On the other hand, a specific lesion to the BBB not involving the CNS may also result in elevated serum levels [30]. Rises in S-100 serum levels are also reported from extracerebral tissues such as marrow, fat or muscle [31]. Despite its obvious lack of specificity, it has nevertheless been found to be an early and sensitive marker of hypoxic brain damage and short-term outcome after cardiac arrest [8,10,11,13-16,19]. Our study is in accordance with these previous reports as at several time points patients with bad neurological outcome had significantly higher serum levels in S-100 protein. Remarkably, the prognostic value of S-100 for neurological outcome in this study diminished over time. Therefore, the initial measurement shortly after admission to the hospital was the most valuable within the post-resuscitation period.

Two previous studies have focused on the effect of therapeutic hypothermia on levels of serum S-100B protein in survivors of cardiac arrest [32,33]. Although Hachimi-Idrissi and colleagues [32] observed a mixed population of patients with asystole and ventricular fibrillation (VF), the study by Tiainen and colleagues [33] only included patients with VF, which did not reveal a significant decline of S-100B values in MTH patients. In contrast, Hachimi-Idrissi and colleagues found a significant decline in MTH patients as compared with NT treatment [32]. The decline was even more pronounced in patients with asystole as initial rhythm. Because patients in our investigation predominantly presented with VF as initial rhythm (66.7% in MTH vs 51.6% in NT) our results seem to support the notion of a connection between initial ECG and the influence of MTH on this surrogate marker.

Although we recognised a trend towards higher survival rates as well as improved neurological outcome, we did not detect significant improvements of these two important endpoints in this investigation which contrasts the findings from previous larger studies. The absolute difference of 11.6% more patients with beneficial neurological outcome and 23.6% higher in-hospital survival rate may nevertheless be seen as a testimony for the potency of this intervention, even in a heterogeneous population. However, it has to be acknowledged that in our study patients with rhythms other than VF were also included, which *per se *have lower chances of survival after cardiac arrest [5,6].

We recognise several limitations in the interpretation of our study. First, our results may be influenced by the relatively low number of patients with the possibility of a lack of adequate power to detect statistical differences. However, in prospective studies of cardiac arrest, 68 patients represent a relatively large population. An enlargement of the study population is nevertheless almost impossible because of an almost 100% implementation of therapeutic hypothermia in the participating hospitals today. Patients treated with NT will therefore scarcely be available for recruitment.

Second, although S-100 is frequently referred to as a specific surrogate marker for the severity of hypoxic brain injury, there are other circumstances that may also result in elevated serum levels. Recently, two studies suggested that serum levels may also be elevated in children [34] as well as in adults [35] during sepsis or septic shock, indicating a potential role of infection and inflammation in the release of S-100 protein. Due to the high infection rate in patients receiving MTH in our study, this might have had a certain influence on our results.

Third, the observational nature of the study which precluded formal randomisation may have led to a systemic bias in a way that patients with a bad prognosis may have been withdrawn from extensive hypothermic treatment. Some of the patients might have been actively or passively cooled before admission to the hospital which could have directed the in-hospital caregivers in most cases to proceed with this therapy rather than abolishing it. The latter might also be an explanation for the difference in temperature between MTH and NT patients at baseline.

Finally, although at the time of the study all participating hospitals were at the time of the study employing standardised intensive care therapy, such as low tidal volume ventilation, tight glucose control etc., the multiple centre setup can not exclude minor differences in standard intensive care therapy or application of MTH. Although no differences in demographic data were evident, the favourable CPC in the MTH group might be influenced by treating only patients with a good prognosis with MTH, while others received NT. The collected outcome data 14 days after ROSC have to be seen as medium-term related endpoints which might not necessarily reflect long-term results.

## Conclusions

In recognising these limitations we conclude that in a mixed population of patients with cardiac arrest, MTH had no influence on S-100 serum levels in survivors of non-traumatic out-of-hospital cardiac arrest in the present investigation. The predictive quality of S-100 levels was best on admission but not on later time points during the first five days of hospitalisation.

## Key messages

• In 68 patients after successful CPR, S-100 levels showed comparable serum levels in patients receiving NT as compared with cooled patients.

• S-100 levels on baseline were significantly lower in patients with a good neurological outcome at 14 days after the event in comparison to their peers with adverse outcome.

• MTH did not significantly influence serum levels of S-100 protein in patients surviving non-traumatic out-of-hospital cardiac arrest in this study.

• The predictive quality of S-100 levels was best on admission but not on later time points.

## Abbreviations

BBB: blood-brain barrier; CPR: cardiopulmonary resuscitation; CNS: central nervous system; CPC: cerebral performance categories; HR: heart rate; MAP: mean arterial pressure; MTH: mild therapeutic hypothermia; NT: normothermia; ROSC: return of spontaneous circulation; SCA: sudden cardiac arrest; VF: ventricular fibrillation.

## Competing interests

The authors declare that they have no competing interests.

## Authors' contributions

MD performed the statistical analysis and drafted the manuscript. DB and CS carried out the acquisition of investigated materials. RR participated in the design of the study and its coordination. MF conceived of the study and participated in its design and coordination and helped to draft the manuscript. All authors read and approved the final manuscript.
